# Single dose of diclofenac or meloxicam for control of pain, facial swelling, and trismus in oral surgery

**DOI:** 10.4317/medoral.20925

**Published:** 2015-11-30

**Authors:** Mariana Orozco-Solís, Yazmín García-Ávalos, Celeste Pichardo-Ramírez, Francisco Tobías-Azúa, Juan-Ramón Zapata-Morales, Othoniel-Hugo Aragon-Martínez, Mario-Alberto Isiordia-Espinoza

**Affiliations:** 1 DDS, Departamento de Estomatología, Unidad Médica Familiar No. 02, Instituto Mexicano del Seguro Social, Delegación San Luis Potosí, S.L.P., México; 2 DDS, Departamento de Investigación y Postgrado, Escuela de Odontología, Universidad Cuauhtémoc, plantel San Luis Potosí, S.L.P., México; 3 MSc, PhD, Departamento de Farmacia, División de Ciencias Naturales y Exactas, Universidad de Guanajuato, Guanajuato, México; 4 MSc, PhD, Departamento de Farmacología, Facultad de Medicina, Universidad Autónoma de San Luis Potosí, S.L.P., México; 5 DDS, MSc, PhD, Departamento de Farmacología, Facultad de Odontología, Universidad Autónoma de Baja California, Mexicali, México

## Abstract

**Background:**

Postoperative pain associated with removal of mandibular third molars has been documented from moderate to severe during the first 24 hours after surgery, with pain peaking between 6 and 8 hours when a conventional local anesthetic is used. Dental pain is largely inflammatory, and evidence-based medicine has shown that nonsteroidal anti-inflammatory drugs are the best analgesics for dental pain. The aim of this study was to compare the analgesic, anti-inflammatory and anti-trismus effect of a single dose of diclofenac and meloxicam after mandibular third molar extraction.

**Material and Methods:**

A total of 36 patients were randomized into two treatment groups, each with 18 patients, using a series of random numbers: Group A, was administered 100 mg of diclofenac; and Group B, 15 mg of meloxicam. Drugs were administered orally 1 hour prior to surgery. We evaluated pain intensity, analgesic consumption, swelling, as well as trismus.

**Results:**

The results of this study showed that patients receiving 15 mg of meloxicam had less postoperative pain (*P*=0.04) and better aperture than those receiving 100 mg of diclofenac (*P*=0.03). The meloxicam group presented less swelling than diclofenac group; however, significant statistical differences were not observed.

**Conclusions:**

Data of this double-blind, randomized, parallel-group clinical trial demonstrated that patients receiving 15 mg of preoperative meloxicam had a better postoperative analgesia and anti-trismus effect compared with who were given 100 mg of diclofenac after third molar extractions.

**Key words:**Diclofenac, meloxicam, dental pain, trismus, third molar surgery.

## Introduction

In the majority of cases, removal of third molars will lead to a significant degree of tissue trauma, causing an inflammatory reaction. The patient develops the common postoperative symptoms and signs of pain, facial swelling, dysfunction, and limited mouth opening (trismus). The pain is typically brief and will peak in intensity in the early postoperative period. Facial swelling and trismus will reach their characteristic maximum 48 to 72 h after surgery (1).

Evidence-based medicine has shown that Non-steroidal anti-inflammatory drugs (NSAID) comprise the best analgesic for dental pain (2). The effects of NSAID are the result of inhibiting Cyclo-oxygenase (COX) enzymes, which catalyze the conversion of arachidonic acid into prostaglandins, which are fatty acids involved in the generation of pain, inflammation, and fever (3). Diclofenac is a commonly prescribed NSAID with analgesic, anti-inflammatory, and antipyretic properties that has been widely used in pain control with good effectiveness after third molar surgery (4,5). However, traditional NSAID such as diclofenac - have been widely used to treat pain, but their long term use is limited by serious gastrointestinal side effects (6). A meta-analysis clearly showed that high-doses of diclofenac (75 mg twice daily) entails similar vascular risks to the average coxib regimen studied. Absolute excess risks were small but serious: compared with placebo, allocation to a coxib or to diclofenac caused around three additional major vascular events per 1,000 participants annually, with one such event causing death (6,7). Meloxicam is a NSAID of the Oxicam class with selectivity toward COX-2 compared with COX-1. It is widely used in the treatment of osteoarthritis, rheumatoid arthritis, ankylosing spondylitis, and other rheumatological conditions (8). In oral surgery, there are few studies concerning the use of meloxicam (8-14).

The aim of this pilot study was to compare the analgesic, anti-inflammatory and anti-trismus effect of a single dose of diclofenac and meloxicam after mandibular third molar extraction.

## Material and Methods

- Design

This study was designed as a double-blind, randomized, parallel-group clinical trial, and was conducted in accordance with the Declaration of Helsinki. The Ethics Committee approved the study design. All of the subjects were informed of possible risks of oral surgery and experimental treatments, and they signed an institutionally approved consent form.

- Selection criteria

Inclusion criteria were age 18-30 years, either sex, free of systemic disease, clinical and radiographic diagnosis of a mandibular impacted third molar with no pain associated up to the day of the surgery, and difficulty of extraction (grade II, III, or IV). Exclusion criteria included use of analgesics 1 week before the procedure, gastritis, peptic ulcer, pregnancy or lactation, and known hypersensitivity to the medications used.

- Randomization and blinding 

**Figure 1:**
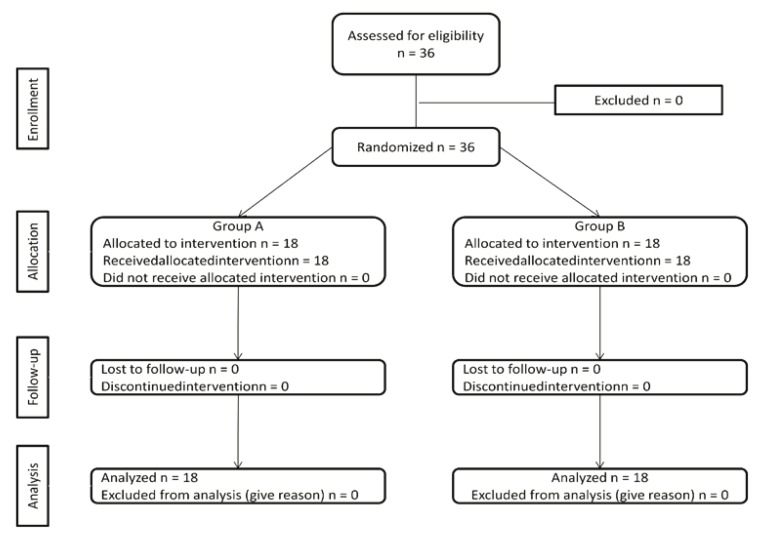
Algorithm showing the progress of subjects through the trial phases. All patients completed the study.

A total of 36 patients from the Department of Maxillofacial Surgery of the School of Dentistry at Cuauhtémoc University, San Luis Potosí, México, were recruited. The patients were randomized into two treatment groups, each with 18 patients, using a series of random numbers: Group A, was administered 100 mg of diclofenac; and Group B, 15 mg of meloxicam. Drugs were administered orally 1 hour prior to surgery. The algorithm is shown in (Fig. 1). Moreover, both patients and an independent evaluator were blinded regarding the treatment administered.

- Interventions

After the administration of the assigned treatment, the tooth was removed. All of the surgical procedures were carried out at the Department of Oral and Maxillofacial Surgery by the same surgeon. Anesthesia was through block of the lingual, buccal, and inferior alveolar nerves using two 1.8-mL capsules of 2% lidocaine-containing 1:100,000 epinephrine. Once anesthesia was administered, surgery was started. A mucoperiosteal flap was prepared by making a distal incision to the mandibular second molar along the anterior edge of the ascending ramus of the mandible. This flap was utilized to close the surgical wound. Suturing was done with 4-0 silk, and the number of sutures was documented. Difficulty of extraction was based on a modified Parant scale as follows: Grade I, extraction with forceps and elevators; Grade II, extraction by osteotomy; Grade III, extraction by osteotomy and coronal section; and Grade IV, extraction by osteotomy, root, and coronal section (13). In all cases, the duration of the surgical procedure (from incision to final suture) was recorded. In each patient, a partial impacted mandibular third molar was extracted.

- Assessment

The patients were administered 500 mg acetaminophen tablets and were instructed to take one or two pills for post surgical medication at least 6 hours apart, according to their requirements. Time of first rescue analgesic medication, i.e., time from the end of surgery until first intake of acetaminophen necessary for the patient, was registered. At the end of the evaluation period (24 hours), the patients returned the unused tablets. The pills were counted to determine the number of pills consumed. Patients having no pain relief 30 min after taking 2 acetaminophen tablets orally were given a 30 mg tablet of sublingual ketorolac as rescue analgesic procedure due to therapeutic failure. Total analgesic consumption (oral acetaminophen and sublingual ketorolac) was evaluated.

A 10 cm Visual Analog Scale (VAS) was employed to assess pain. The VAS consisted of an interval scale ranging from 0, representing no pain or discomfort, to 10, representing maximal pain or discomfort. The VAS report was recorded each hour for 12 hours after completion of surgery, and the last evaluation was conducted at 24 hours.

Facial swelling was evaluated by the method used by Ustun *et al*., (2003) (15). Distances between angle of the mandible and four different anatomical facial points (soft-tissue pogonion, outer corner of mouth, and lateral corner of eye and tragus) were assessed. In the same manner, distances of tragus to three anatomical facial points (soft-tissue pogonion, outer corner of mouth, lateral corner of eye) were evaluated. Trismus was assessed by measuring the maximal aperture. Both facial swelling and trismus were evaluated preoperatively and at 6, 24, 48, and 72 hours, and a final evaluation was performed on day 7. Intra- and postoperative complications as well as adverse events were recorded.

A standardized independent evaluator measured these parameters (pain, facial swelling, and trismus) at each time point.

- Statistical methods

Data were expressed as mean and Standard Deviation, medians and ranges, or frequencies. For numerical variables with normal distribution, the Student t test was used, while for numerical variables without normal distribution and ordinal variables, the Mann-Whitney U test was utilized. For categorical variables, the Fisher exact test was employed. A *p* value <0.05 was considered a significant statistical difference.

## Results

A total of 36 patients were enrolled in the study, and all patients were included in the statistical analysis. There were no statistically significant differences between the study groups with regard to the number of patients included and their age distribution, sex, height, and weight. Surgical variables that could have influenced postoperative pain intensity were considered homogeneous between the groups, including length and difficulty of the surgical procedure ( Table 1).

**Table 1:**
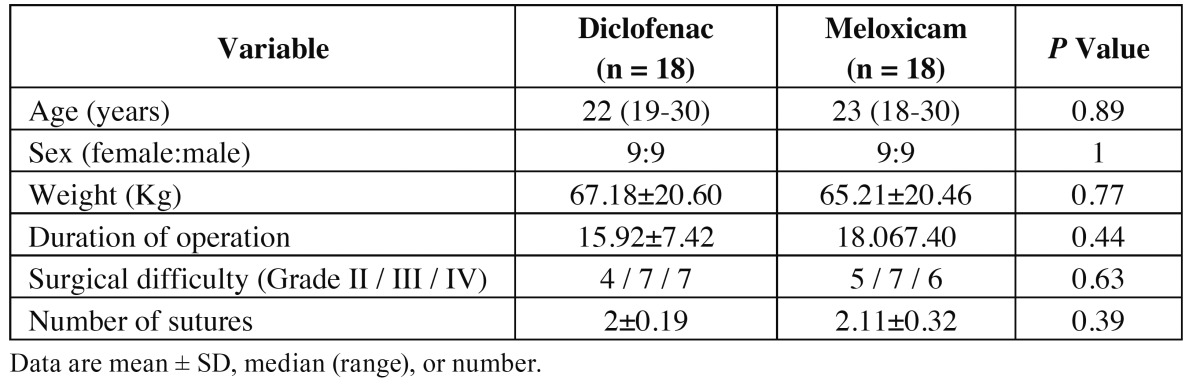
Demographic and surgical characteristics.

Time of first rescue analgesic medication, the number of patients taking acetaminophen at 3, 6, 9, 12 and 24 hours, analgesic consumption, and the number of patients requiring rescue analgesic procedure with sublingual ketorolac due to therapeutic failure was similar in the two treatment groups ( Table 2).

**Table 2:**
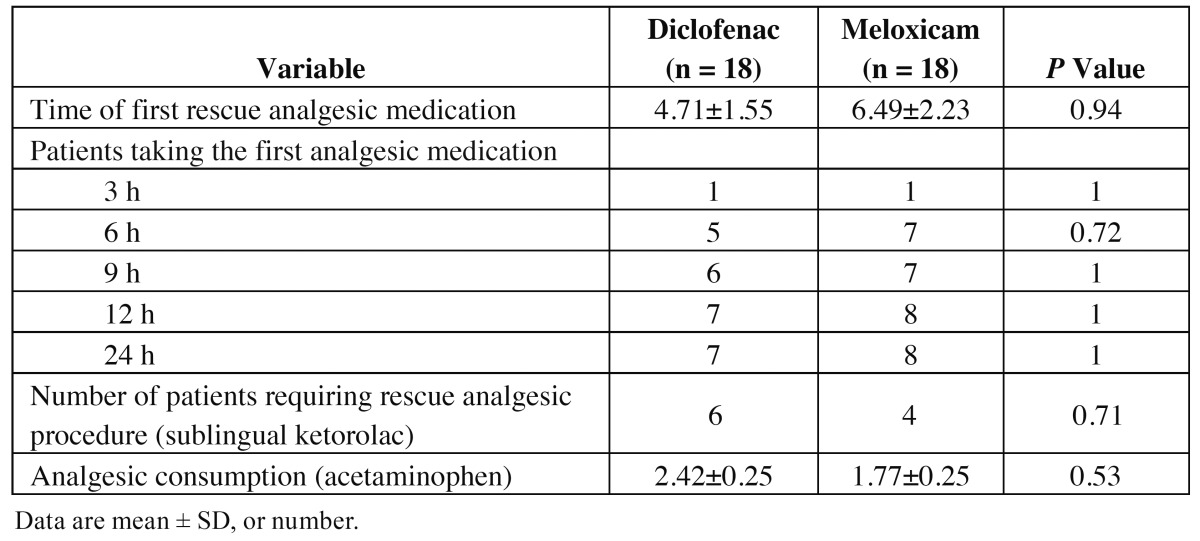
Comparison of analgesic efficacy of diclofenac and meloxicam.

**Figure 2:**
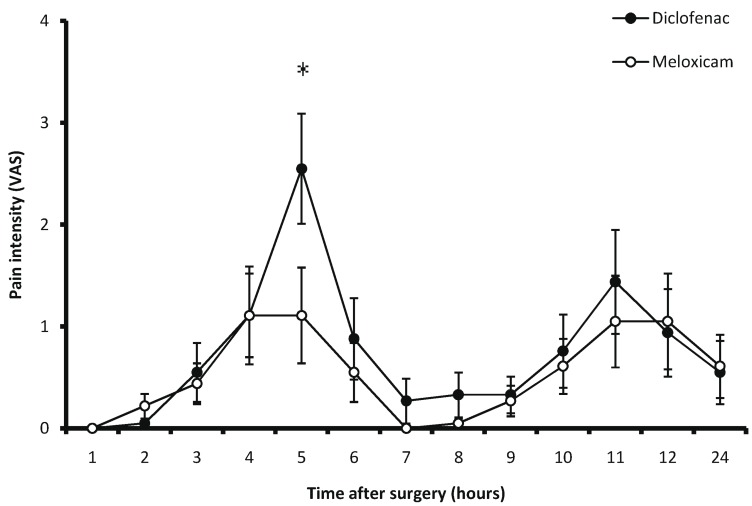
Pain intensity evaluated by the VAS during 24 postoperative hours (*P=0.04).

Pain levels assessed by the VAS had their peaks at fifth hour postoperative and then began to decrease. Meloxicam was only better than diclofenac at fifth hour postoperative according to VAS scores (*P*=0.04), (Fig. 2).

As mentioned previously, seven facial anatomical distances, measured pre- and post-surgery, were employed to determine facial swelling and the possible anti-inflammatory effects of the medications tested. However; no statistical differences were observed ( Table 3).

**Table 3:**
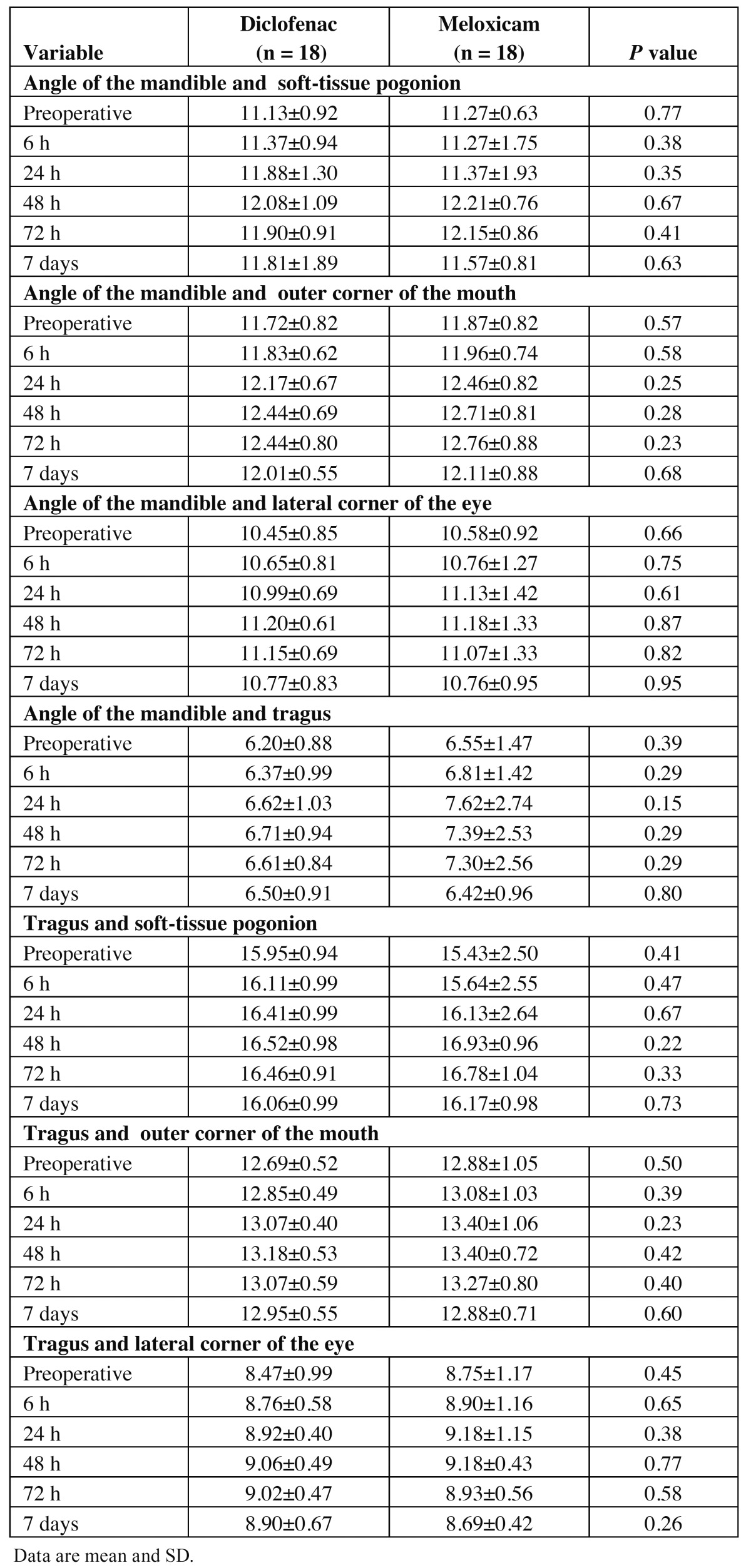
Facial swelling throughout the period of evaluation.

**Figure 3:**
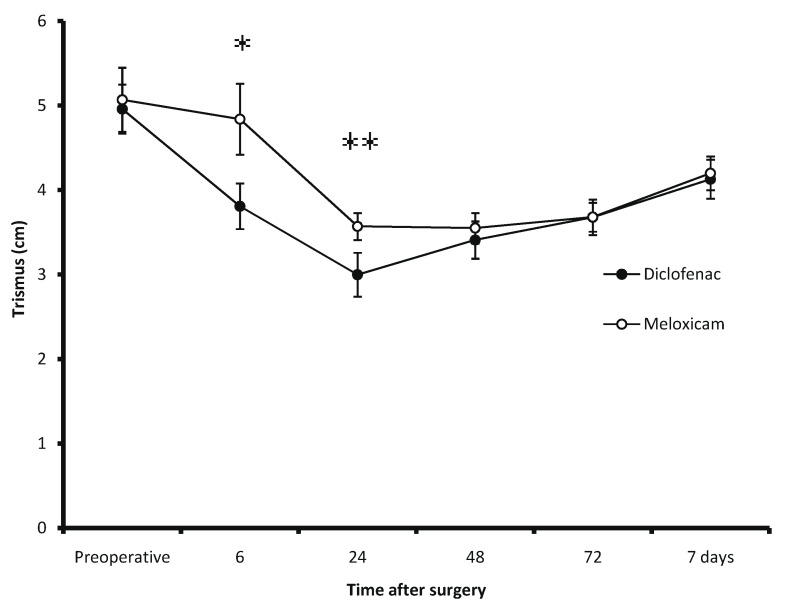
Evaluation of trismus (*P=0.03; and **P=0.04).

In both treatment groups, aperture before surgery was 5 cm and after surgery; this decreased about 40% at 24 hours post operatively and, incrementing as time went on. Trismus scores were superior in patients who took meloxicam when compared with those patients receiving diclofenac (Fig. 3).

Finally, there were no complications associated with the surgical procedure itself, and none of the patients reported adverse events associated with the medications.

## Discussion

This clinical trial evaluated the efficacy of a single dose of diclofenac and meloxicam administered by oral route for control of pain, inflammation and trismus after a mandibular third molar surgery. Duration of analgesia for diclofenac was in agreement with previous reports (16,17). However, the duration of analgesia of meloxicam for mandibular third molar surgery with osteotomy was lower compared with the sole report in the literature (10). A more intense pain peak was observed at fifth hour postoperative. Second peak of postoperative pain was observed at 11 hours. It is important to observe that pain control was nearly similar for both treatment groups throughout the evaluation period according to the VAS scores, except on the fifth hour, on which a statistical difference was observed in meloxicam’s favor. Assessment of facial swelling employing the anatomical facial points was analogous for the both groups. It reached its peak between 48 and 72 hours after surgery. Both postoperative pain and facial swelling were in agreement with those previously reported in the literature (1). Meanwhile, trismus reached its greatest severity at 24 hours after surgery. Patients taking meloxicam had an aperture larger than those patients receiving diclofenac. We consider that these differences with respect to pain intensity and mouth opening in meloxicam’s favor can be of clinical importance. The trend of data of this study could be confirmed with a clinical trial including a large sample of patients and a superior surgical difficulty comparing the analgesic, facial swelling and anti-trismus effects of meloxicam and diclofenac after third molar surgery.

The practice of initiating administration of analgesic drugs preoperatively is particularly controversial. The current tendency is to start treatment at an earlier stage than in the past, because it has been shown that postoperative pain can be avoided almost entirely through analgesic pre medication (18). In our case, all surgeries implied a degree of osteotomy and it was assumed that all patients would present postoperative pain. Medication was provided on a prophylactic basis - prior to the appearance of pain, and on ethical grounds.

One of the problems of evaluating the analgesic, anti-inflammatory and anti-trismus efficacy of a drug is related to the pharmacokinetics and pharmacodynamics of the drugs. Another important factor to be taken into account is the evaluation period. In effect, with the introduction of long-acting analgesics, prolonged observation periods have become necessary in the context of single dose studies (19).

The analgesic effect of both diclofenac and meloxicam has been shown in several previous clinical studies in comparison with others NSAID in third molar surgery. Diclofenac has shown a similar analgesic effect to that of paracetamol (20,21), ibuprofen (18,20), and ketorolac (22). However, aceclofenac (23), lornoxicam (24), and tenoxicam (25) have shown better analgesic efficacy than diclofenac in this type of surgery. Sener *et al*., (2005) carried out a random, single-blind study to compare the analgesic efficacy of diflunisal, naproxen, meloxicam, acetaminophen and rofecocixib in oral surgery. Nevertheless, the authors found no differences related with analgesic efficacy or adverse effect (9). Before our study and to our knowledge, meloxicam had only been used in comparison with NSAID in two multiple-dose studies on oral surgery. A clinical trial by De Menezes and Cury (2010) employing nimesulide 100 mg for 5 days in comparison with meloxicam 7.5 mg twice daily for 5 days demonstrated an adequate and similar analgesic effect of both drugs. Nonetheless, nimesulide was more effective than meloxicam in the control of swelling and trismus following third molar extraction (11). In our study, meloxicam was better in postoperative pain management and trismus than diclofenac. Meanwhile, our results on facial swelling were similar for both drugs.

In this study no patients reported adverse effects in relation to the use of these drugs. Nevertheless, clinical trials using diclofenac in third molar surgery have reported adverse effects over the digestive and nervous system (20,23,24). Moreover, meloxicam has been associated with mild adverse effects in third molar surgery. Sener *et al*., (2005) reported nausea, vomiting, bleeding in surgical site, allergy, and gastrointestinal symptoms when meloxicam was used in oral surgery (9).

In conclusion, data of this pilot study suggest that the preoperative administration of a single dose of meloxicam produces superior postoperative analgesia and anti-trismus effects compared with diclofenac after mandibular third molar extraction.
